# Reproductive pattern and population dynamics of commercial red swamp crayfish (*Procambarus clarkii*) from China: implications for sustainable aquaculture management

**DOI:** 10.7717/peerj.6214

**Published:** 2019-01-23

**Authors:** Shiyu Jin, Lisa Jacquin, Mantang Xiong, Ruojing Li, Sovan Lek, Wei Li, Tanglin Zhang

**Affiliations:** 1 State Key Laboratory of Freshwater Ecology and Biotechnology, Institute of Hydrobiology, Chinese Academy of Sciences, Wuhan, China; 2 University of Chinese Academy of Sciences, Beijing, China; 3 Laboratoire Evolution et Diversité Biologique (EDB), UMR 5174, Université de Toulouse, CNRS, IRD, UPS, Toulouse, France

**Keywords:** *Procambarus clarkii*, Spawning period, Population growth, Mortality and exploitation rates, Sustainable management

## Abstract

**Background:**

The red swamp crayfish, *Procambarus clarkii* (Girard, 1852), is one of the most promising freshwater species for aquaculture in China. Understanding its reproductive pattern and population dynamics is crucial for sustainable management, but there is currently a lack of fundamental knowledge of commercial *P. clarkii* populations. Therefore, the purpose of this study was to investigate the reproductive pattern and population dynamics of commercial *P. clarkii* throughout the yearly cycle.

**Methods:**

A total of 2,051 crayfish (1,012 females and 1,039 males) were collected from March 2016 to February 2017 in the area of Selection and Reproduction Center of Crayfish. The reproductive pattern was evaluated by the gonadosomatic index (GSI), hepatosomatic index (HSI), ovarian development and fecundity. Growth, mortality rates and exploitation rate were estimated by electronic length frequency analysis by R package “TropFishR” based on data of cephalothorax length (CTL).

**Results:**

Our results demonstrated that spawning activities of *P. clarkii* took place from September to November, with a mean fecundity of 429 ± 9 eggs per female. There were two recruitments yearly, a major one from October to November and a minor one from March to May. With respect to population growth, five growth cohorts were identified for both females and males. Crayfish grew faster but attained smaller asymptotic maximum CTL as indicated by higher growth coefficient (*K*), growth parameter index (*Ø*′) and lower asymptotic CTL (*L_inf_*). The estimates of total mortality rate (*Z*), natural mortality rate (*M*) and fishing mortality rate (*F*) were 1.93, 1.02, 0.91 year^−1^ for females and 2.32, 0.93, 1.39 year^−1^ for males, which showed that the mortality of male crayfish was mainly caused by fishing. The estimates of exploitation rate (*E*) indicated that male crayfish were overexploited, with the values of 0.47 and 0.60 year^−1^ for females and males, respectively.

**Discussion:**

*P. clarkii* spawned from September to November while two recruitments were observed yearly. We inferred that some eggs, prevented from hatching by low water temperature in winter, were more likely to hatch in the next spring. Moreover, the fishing mortality rate was relatively high for males, which might be related to the males-directed selection during the reproductive period. The higher values of exploitation rate in our study confirmed that males *P. clarkii* were overexploited and were under high fishing pressure. We thus suggest reducing fishing intensity on immature crayfish and avoid sex selection during the reproductive period to improve the overall sustainability of commercial *P. clarkii* populations.

## Introduction

Aquaculture has become a multinational industry over the last 30 years and is expected to maintain an average annual growth rate of 44% over the period 2010–2030 (FAO, 2017). Currently, it has been the fastest growing food-producing sector and has supplied more than 50% of global aquatic food consumption in the world (Wang et al., 2015). In 2016, global aquaculture production reached approximately 80 million tons, corresponding to $232 billion in sales (FAO, 2018). Among commercially farmed species, the red swamp crayfish *Procambarus clarkii* (Girard, 1852), was the second most produced species accounting for 12% of total crustaceans aquaculture production (FAO Yearbook, 2018). China, the top-ranking aquaculture country, has undergone remarkable development in its culture. *P. clarkii*, originally distributed in northeastern Mexico and the south-central United States, has been introduced into Nanjing, China from Japan since the late 1930s (Henttonen & Huner, 1999; Hobbs, Jass & Huner, 1989; Shu & Ye, 1989; Li et al., 2012). The crayfish displays an r-strategy, exhibiting short life cycles and rapid growth, and they tolerate poor environment conditions (Cruz & Rebelo, 2007). Now it can be found in various freshwater habitats such as rivers, ponds, rice fields and ditches of most provinces in China (Fisheries Department of the Chinese Ministry of Agriculture, 2017). Although its fast spread was reported to reduce the diversity of plankton, invertebrates, and tadpoles (Zhang et al., 2003; Wu et al., 2008; Zhang et al., 2014), the huge commercial values created great incentives for farmers to the culture of *P. clarkii*. The production has achieved 852,300 tons in 2016 and represented 41.94% of China freshwater shrimp aquaculture (Fisheries Department of the Chinese Ministry of Agriculture, 2017). However, the growing demand intensifies immense fishing pressure on commercial populations, which results in population depletion and slow recovery rates (Naylor et al., 2000; Tidwell & Allan, 2001). Therefore, effective fishery management efforts are now needed to alleviate fishery crises and promote commercial *P. clarkii* populations’ sustainability. Correspondingly, fisheries management should be based on a better understanding of population life-history characteristics, which are supposed to induce changes in management policies.

Reproduction, growth, and mortalities are the most important life-history parameters for population maintenance, and studies on these parameters are thus crucial for fishery management (Fatemi et al., 2009; He et al., 2011). Recently, efforts have been made to assess the status of reproduction and population dynamics of *P. clarkii* populations in wild, but little information is available on population characteristics under commercially-cultured conditions. Previous studies showed that *P. clarkii* displayed considerable plasticity and variability in reproductive patterns in different regions of the world. For example, some authors reported that the reproduction of *P. clarkii* had a clear annual periodicity, with most spawning events confined to autumn in different locations such as USA (Oluoch, 1990), Germany (Chucholl, 2011), and Italy (Dörr et al., 2006). While others argued that there existed two or more spawning periods yearly for *P. clarkii* in Portugal (Sousa et al., 2013), Italy (Scalici & Gherardi, 2007), Kenya and Spain (Gutiérrez-Yurrita et al., 1999; Gutiérrez-Yurrita & Montes, 1999). In China, authors also reported different results. For example, the population was proved to spawn once yearly in Poyang lake (Jiangxi province, subtropical climate with annual mean precipitation of 1996 mm and annual mean temperature of 18.9 °C, Xiao et al., 2011), Huangjin Lake (Wuhan, Hubei province, subtropical climate with annual mean precipitation of 1,236 mm and annual mean temperature of 17.2 °C, Lv, 2006; Gong et al., 2008), and Xuyi (Jiangsu province, transitional zone between temperate and subtropical climate with annual mean precipitation of 972 mm and annual mean temperature of 15.3 °C, Xu et al., 2014) while twice a year in Wuhan (Hubei province, Dai et al., 2008).

In fish or crayfish population dynamics studies, understanding of population parameters such as growth (growth coefficient *K* and growth parameter index *Ø*′), mortalities (total mortality rate *Z*, natural mortality rate *M*, and fishing mortality rate *F*) has important implications for population assessment (Rochet et al., 2000). Estimates of these parameters provide fundamental information for predicting population growth and developing sustainable exploitation strategies (Nurul Amin, Zafar & Halim, 2008; Ochwada-Doyle et al., 2014).

Usually, growth parameters such as *K* and *Ø*′ are used for evaluation of growth performance under a variety of environmental stresses such as under aquaculture conditions (Pauly, 1991; Živkov, Trichkova & Raikova-Petrova, 1999). Quantitative assessment of mortality is also a significant step to improve our understanding of population dynamics. *M* was defined as the mortality caused by all possible causes except fishing and it could be obtained from the values of *Z* minus *F* (Pauly, 1980). *M*, *Z*, and *F* are thus crucial parameters that are commonly used in fisheries assessment and management, but they are poorly known for commercial *P. clarkii* populations (Kenchington, 2014; Nadon et al., 2015; Williams et al., 2015). Moreover, for successful fisheries management, it will be necessary to further examine the exploitation states for different populations. The previous studies suggest that a value of 0.5 for *E* represents the optimum exploitation condition while a value of *E* > 0.5 points toward over-fishing (Gulland, 1971; Clasing et al., 1994).

Up to date, characteristics of those population parameters have been extensively studied on *P. clarkii* wild populations from Europe, with great emphasis on the prevention of further invasions in Italy (Scalici & Gherardi, 2007; Scalici et al., 2010; Dörr & Scalici, 2013; Maccarrone et al., 2016; Donato et al., 2018), France (Coignet, Pinet & Souty-Grosset, 2012; Meineri et al., 2014), Germany (Chucholl, 2011), Portugal (Anastácio et al., 2009), and Spain (Alcorlo, Geiger & Otero, 2008). Nevertheless, few studies have been conducted on commercially cultured *P. clarkii* populations. Despite its high commercial importance in China, knowledge of reproduction and length-based population dynamics information, including growth, mortalities and exploitation rate of commercially cultured populations is generally limited. There is, thus, a need to target those biological characteristics of commercial populations for successful aquaculture management.

The objective of the present study was to evaluate the reproduction, growth, mortalities and exploitation rate of the commercial *P. clarkii* population in China. For this purpose, we studied: (1) reproductive pattern of females by measuring the GSI, HSI, ovarian development, and fecundity; and (2) population dynamics by estimating growth (*K* and *Ø*′), mortality rates (*Z*, *M*, and *F*) and exploitation rate (*E*). Our work will hopefully provide background information to develop effective and sustainable management strategies of *P. clarkii* commercial populations.

## Materials & Methods

### Study area

The study is carried out in the Selection and Reproduction Center of Crayfish (30.41°N, 112.75°E), Qianjiang, which is recognized as the land of red swamp crayfish in China by the Ministry of Agriculture of the People’s Republic of China. This region extends over 200 ha and encloses many artificial ponds.

The studied area has a surface area of 33,350 m^2^, which is under good management and is referred as the model of crayfish culture. In this area was planted *Hydrilla verticillata*, preferred by *P. clarkii* and tolerant to high water temperatures in summer. This submerged macrophyte can provide supplementary nourishment, refuge for crayfish and supports maintaining suitable water quality. Quicklime (15–22.5 grams/m^3^) was used monthly to prevent diseases and eradicate other unwanted aquatic organisms (e.g., silver carp, rice field eel, gold fish, and loach). Crayfish stocking was from March to April, with individual sizes ranging from 3 to 5 g. The stocking density was 15 individuals/m^2^. Two commercial diets were commonly used as a main food source for crayfish during the study period and were purchased from Charoen Pokphand Group (WHS001-2016, diet 1: 30.23% crude protein, 10.74% crude lipid, 10.18% moisture, and 8.70% ash; diet 2: 26.53% crude protein, 10.41% crude lipid, 13.96% moisture, and 6.87% ash). From March until May crayfish were fed with high protein level pellets (diet 1) in order to reach commercial sizes in a short period. In the pond feeding rates differed in time, but were in general about 3% of the crayfish biomass per day.

During the sampling period, the annual mean water temperature was 19.75 °C, ranging from 8.65 °C in January to 31.25 °C in August. The water depth was 1–1.5 m. Other water physical-chemical parameters were: pH 8.61–9.30; ammonia nitrogen 0.14–0.43 mg/L; nitrite 0.15–0.25 mg/L; total nitrogen 1.06 ± 1.67 mg/L; total phosphorus 0.0445 ± 0.17 mg/L; chemical oxygen demand (to quantify the amount of oxidizable pollutants in https://en.wikipedia.org/wiki/Water) 5.83–8.80 mg/L; and chlorophyll-a 14.55–31.67 µg/L.

### Crayfish sampling

Crayfish were collected monthly from March 2016 to February 2017 with 8 cylindrical traps baited with fresh silver carp. The traps were 100 cm long with 5 mm mesh, 30 cm cross-section, and two opposing funnels 10 cm in diameter. During each sampling event, trapping was performed and retrieved in the afternoon. The periods of trapping were one day from June to September; two days from March to May, and October; and three days from November to February. The same sampling site order and timetable were followed every month in order to minimize the bias in measurement. Catch per unit effort (CPUE) was calculated for each sampling as the daily number of crayfish per trap.

Sampled crayfish were sorted by sex. Cephalothorax length (CTL, from the tip of the rostrum to the cephalothorax posterior margin) was measured by a 0.01 mm precision caliper. Crayfish weight was determined by a 0.01 g precision scale. All samples were then transported to the laboratory to dissect. During the whole sampling period, water temperature was recorded every two hours by a HOBO data-logger (UA-002-64, HOBO Pendant temperature/light 64 K data logger Onset, Bourne, MA, USA).

### Reproductive pattern analysis

After transporting to the lab, females were checked for attached eggs, if present, they were counted to determine the fecundity. Then they were frozen to −20 degrees to dissect, following the European Directive 2010/63/EU for animal experiments. The gonads and hepatopancreas of females were weighted to calculate the gonadosomatic index (GSI) (measuring the sexual maturity and relating to ovary development) and hepatosomatic index (HSI) (indice of energy status):

\begin{eqnarray*}& & \text{GSI}=100\times {W}_{g}/{W}_{t} \end{eqnarray*}

\begin{eqnarray*}& & \text{HSI}=100\times {W}_{h}/{W}_{t} \end{eqnarray*}

Where *W*_*g*_, *W*_*h*_, and *W*_*t*_ are the gonad weight, hepatopancreas weight and body weight of *P. clarkii*, respectively.

Dissected gonads were fixed for 24 h in Bouin’s solution (Wuhan Servicebio Technology Company, Wuhan, China) for histological analysis. Samples were dehydrated in 50%, 70%, 85%, 90%, 95%, and 100% ethanol and embedded in paraffin block. Then they were subjected to microtomy to obtain sections with 4 µm (Leica RM2016, USA). Slides were deparaffinized (2 changes of xylene, 20 min each; 3 changes of 100% ethanol, 5 min each), rinsed in distilled water. Then all the slides were stained with hematoxylin and eosin (Kiernan, 1999; Suvarna, Layton & Bancroft, 2012). The histopathological analyses were performed on micrographs under an Olympus BX53 microscope (Fig. S1 ). The ovarian development was classified into seven stages: stage I, stage II, stage III, stage IV, stage V, stage VI, stage VII, following the method described by the previous study (Kulkarni, Glade & Fingerman, 1991).

### Population dynamics parameters estimates

In order to estimate the population dynamics parameters (*K*, *L*_*inf*_, *Ø*′, *Z*, *M*, *F*, and *E*), the CTL data for each sex was used because it was more reliable in contrast to the flexible abdominal joint of crayfish (Ghia et al., 2015). *K* is referred to a relative growth rate and has dimensions of time^−1^ and *Ø*′ has a clear biological meaning (the intercept of log*K* and log *L*_*inf*_ regression) and it is used to compare seasonal estimates of growth parameters as well as overall estimates by different fitting techniques (Al-Hosni & Siddeek, 1999).

To estimate these parameters, we used the electronic length frequency analysis (ELEFAN), a system of fishery assessment procedures that is commonly employed to estimate population parameters based on length-frequency data (Pauly & David, 1980; Taylor & Mildenberger, 2017). The FISAT software has been the most frequently used for estimating population parameters. However, it is limited in importing data and performing automated analyses (Mildenberger, Taylor & Wolff, 2017). The R package “TropFishR” remedies these shortcomings and uniquely adds the further data-limited method capacity by including traditional and updated ELEFAN method (two optimization approaches: generalized simulated annealing ELEFAN_SA, and genetic algorithm ELEFAN_GA) for growth curves fitting and parameters estimates (Mildenberger, Taylor & Wolff, 2017; Taylor & Mildenberger, 2017). So in this study, the frequency distributions were analyzed and fitted with growth curves by the ELEFAN of R package “TropFishR”.

The parameters were calculated as follows:

*Ø*′ = log*K* + 2log *L*_*inf*_ (Pauly & Munro, 1984);

The expected longevity (*t*_*max*_): *t*_*max*_ = 3∕*K* + *t*_0_ (Huang et al., 2012);

The *Z* and *M* were obtained through the Powell-Wetherall method (Wetherall, 1986). The *F* is obtained by subtracting *M* from *Z*. The *E* is defined as *E* = *F*/*Z.*

Where *L*_*inf*_ is the asymptotic CTL (calculated as *L*_*max*_/0.95, where *L*_*max*_ is the maximum recorded CTL); *K* is the growth coefficient; *t*_0_ is the initial condition parameter (when crayfish have CTL = 0, although biologically meaningless, it represents an important component of curve) and can be calculated as *ln*(−*t*_0_) =  − 0.3922–0.2752*lnL*_*inf*_ − 1.308*lnK*.

### Statistical analyses

Because normality and homogeneity of variance assumptions were not satisfied, we used non-parametric Kruskal–Wallis test followed by pairwise Wilcoxon Rank Sum test (post hoc test) to detect the differences in GSI, HSI, CPUE, and the estimated population dynamics parameters. Student’s *t*-test was used to compare the differences of CPUE between females and males. The relationships between CPUE and temperature, and GSI and HSI were analyzed by Pearson’s product-moment correlation test. Chi-squared test was used to access the sex ratio balance among different months. Generalized additive model (GAM) was used to illustrate the relationships between weight, CTL, and cephalothorax width and fecundity. Statistical differences were set to 0.05 and all statistical analyses were performed in the software R version 3.3.2 (R Core Team, 2017).

## Results

### Sampling features

**Figure 1:**
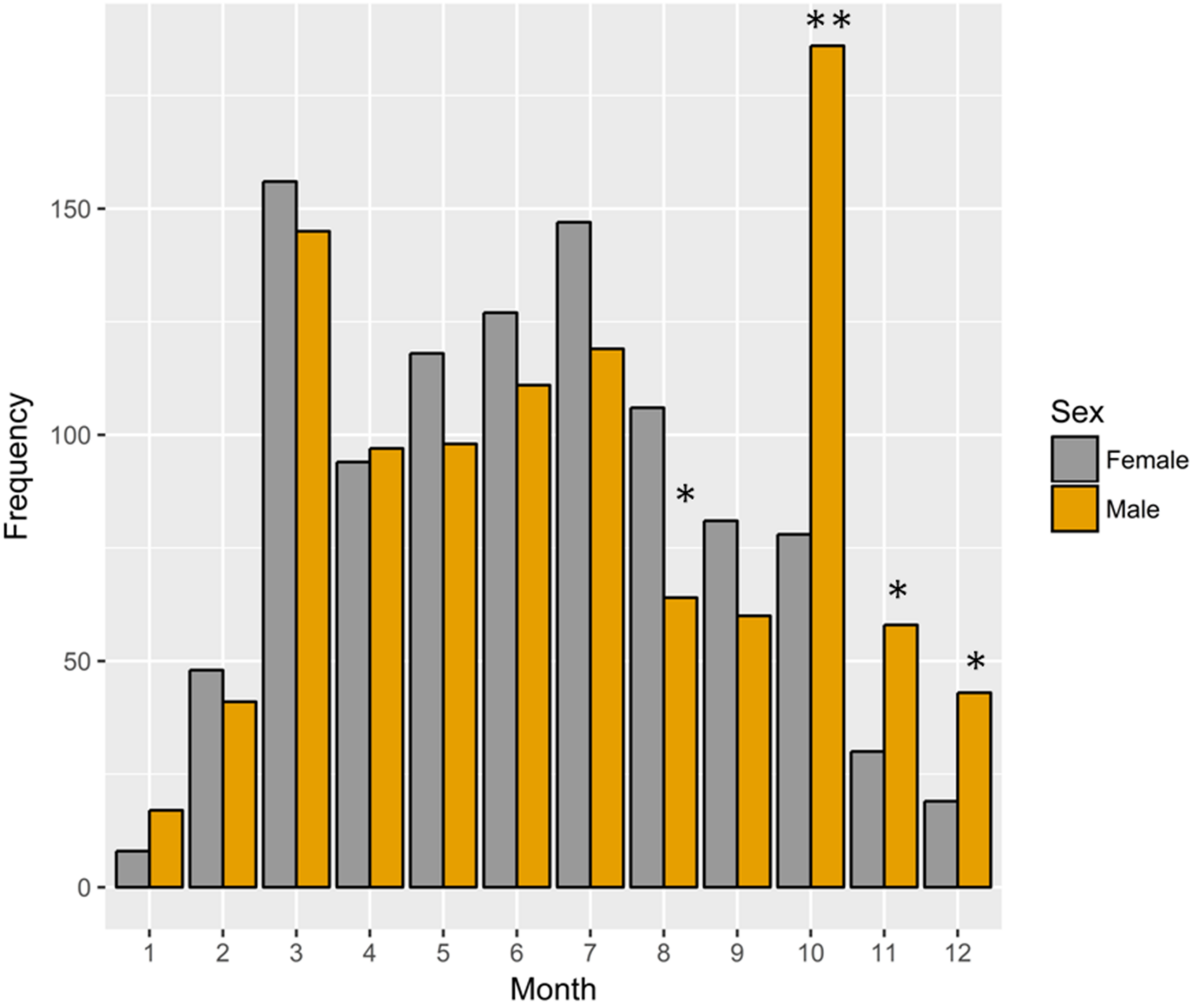
Frequency histogram for females and males of *Procambarus clarkii* throughout the year. Significant differences from expected 1:1 ratio between sexes are shown by asterisks (* *P* < 0.05 and ** *P* < 0.001).

A total of 2,051 individuals (1,012 females and 1,039 males) were captured in the studied area from March 2016 to February 2017. During the entire sampling period, the sex ratio (females/males, Fig. 1) did not differ significantly from expected 1:1 sex ratio (chi-square test: *χ*^2^ = 0.36, *P* = 0.55). However, they showed significant differences from 1:1 from August to December except September, with females abundant in August, and the situation reversed from October to December (chi-square test: August, *χ*^2^ = 10.38, *P* = 0.001; September, *χ*^2^ = 3.13, *P* = 0.080; October, *χ*^2^ = 44.18, *P* < 0.001; November, *χ*^2^ = 8.91, *P* = 0.003; December, *χ*^2^ = 9.29, *P* = 0.002).

### Catch per unit effort (CPUE)

**Figure 2:**
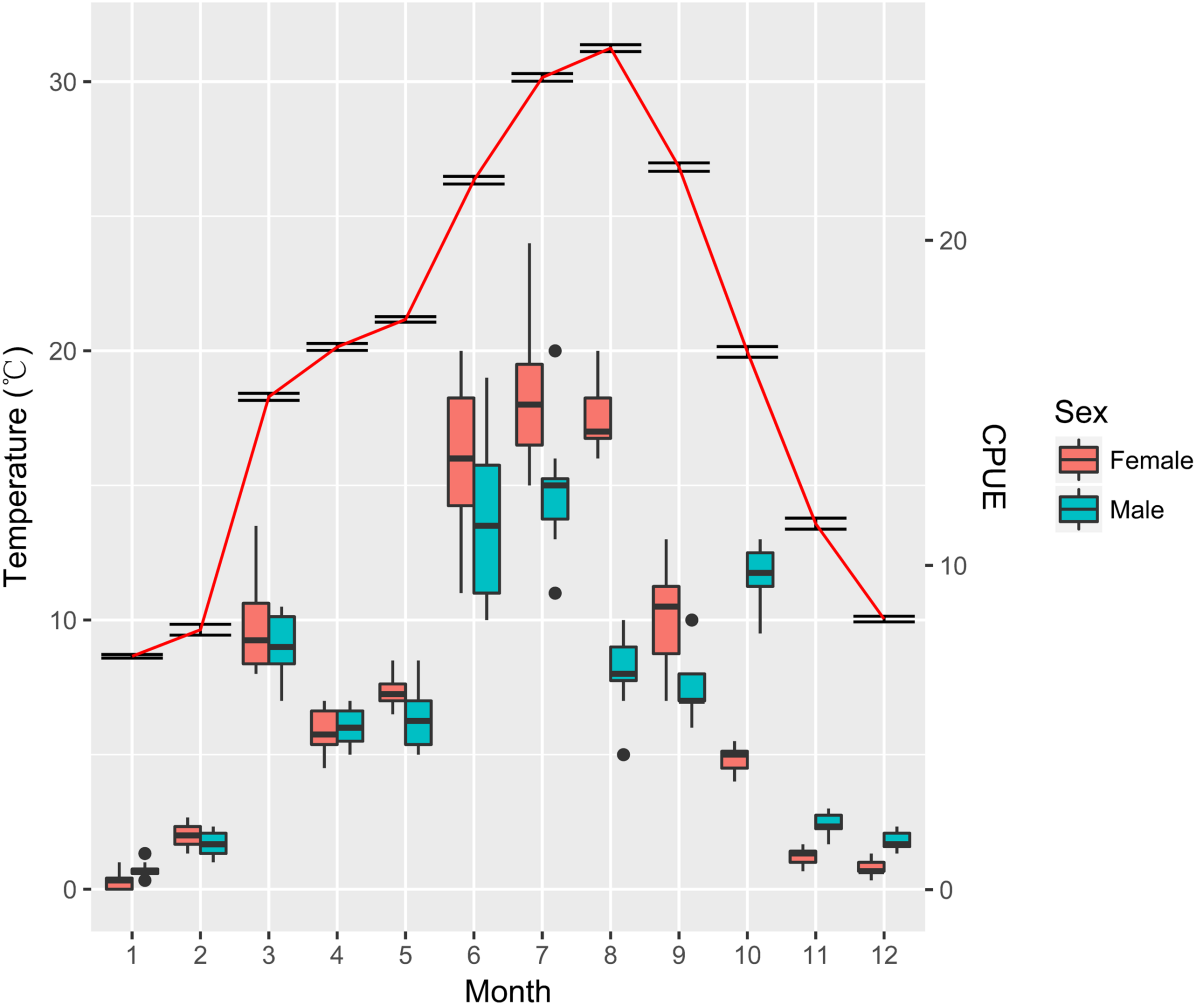
Variation of temperature and catch per unit effort (CPUE) for females and males of *Procambarus clarkii* throughout the year. Box-plot representation: the horizontal line inside the box represents the median, and the lower and upper borders of the box represent the 25th and 75th percentiles, respectively. The upper and lower whiskers indicate the maximum and minimum range of the data excluding outliers. Temperature values are shown as mean ± SE.

The temperature and CPUE of female and male crayfish are shown in Fig. 2. The CPUE were significantly different across months both for females and males (Kruskal–Wallis test, females: *χ*^2^ = 91.34, *P* < 0.001; males: *χ*^2^ = 89.04, *P* < 0.001). Post hoc analyses showed that the CPUE of females in June, July, and August were significantly higher than other months, while for males, July was significantly higher than other months except for June (pairwise Wilcoxon Rank Sum test, females: June-March: *P* = 0.002, June–September: *P* = 0.004, others: *P* < 0.001; males: July–June: *P* = 0.395, others: *P* < 0.05). There were no significant differences observed between the CPUE of females and males (Students’ *t*-test, *t* = 1.97, *P* = 0.052). We further found that there were strong correlations between temperature and CPUE for females and males (Pearson correlation test, females: *r* = 0.93, *t* = 8.25, *P* <0.001; males: *r* = 0.81, *t* = 4.41, *P* = 0.001).

**Figure 3:**
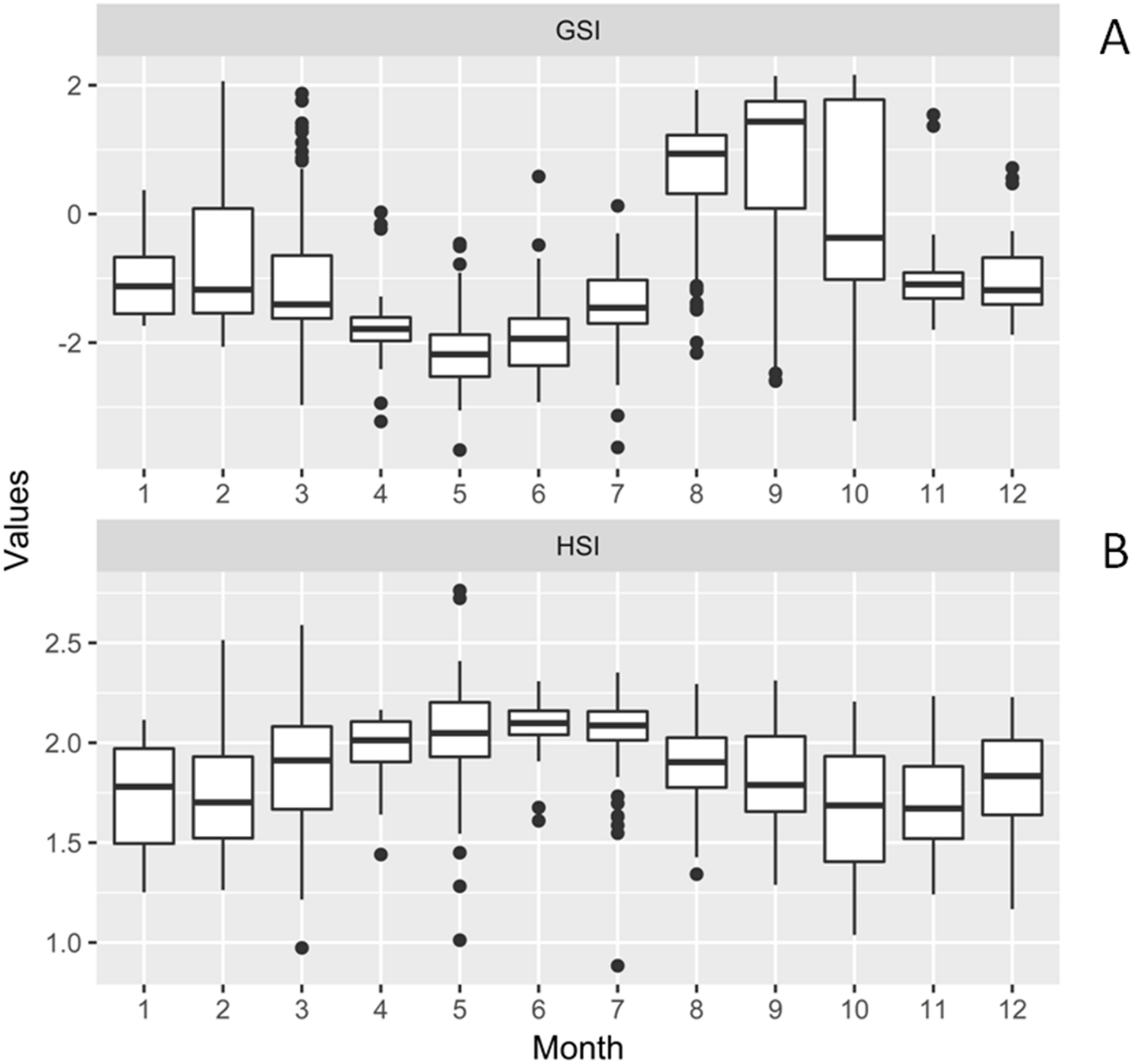
Box-plot of gonadosomatic index (GSI) and hepatosomatic index (HSI) for females of *Procambarus clarkii* during the sampling period. Box-plot representation: the horizontal line inside the box represents the median, and the lower and upper borders of the box represent the 25th and 75th percentiles, respectively. The upper and lower whiskers indicate the maximum and minimum range of the data excluding outliers.

### Reproductive pattern analysis

The monthly variations of GSI and HSI for females are shown in Fig. 3. There were significant differences in GSI throughout months. The GSI in August, September, and October were significantly higher than other months by Kruskal-Wallis and pairwise Wilcoxon Rank Sum test (*χ*^2^ = 369.84, September–January: *P* = 0.003, October–January: *P* = 0.02, others: *P* < 0.001). It increased remarkably from August to September. Although there was slight increase in February, no significant differences were observed, when compared with January, March, November and December (*χ*^2^ = 369.84, January: *P* = 0.70; March: *P* = 0.06; November: *P* = 0.78; December: *P* = 0.80).

The HSI decreased progressively from September to October. Comparisons among different months by Kruskal-Wallis and pairwise Wilcoxon Rank Sum test showed that April, May, June, and July had significantly higher HSI values than that of other months (*χ*^2^ = 266.99, all *P* < 0.05). Furthermore, we found that GSI was negatively correlated with HSI (Pearson correlation test *r* =  − 0.38, *t* =  − 10.79, *P* < 0.001).

**Figure 4:**
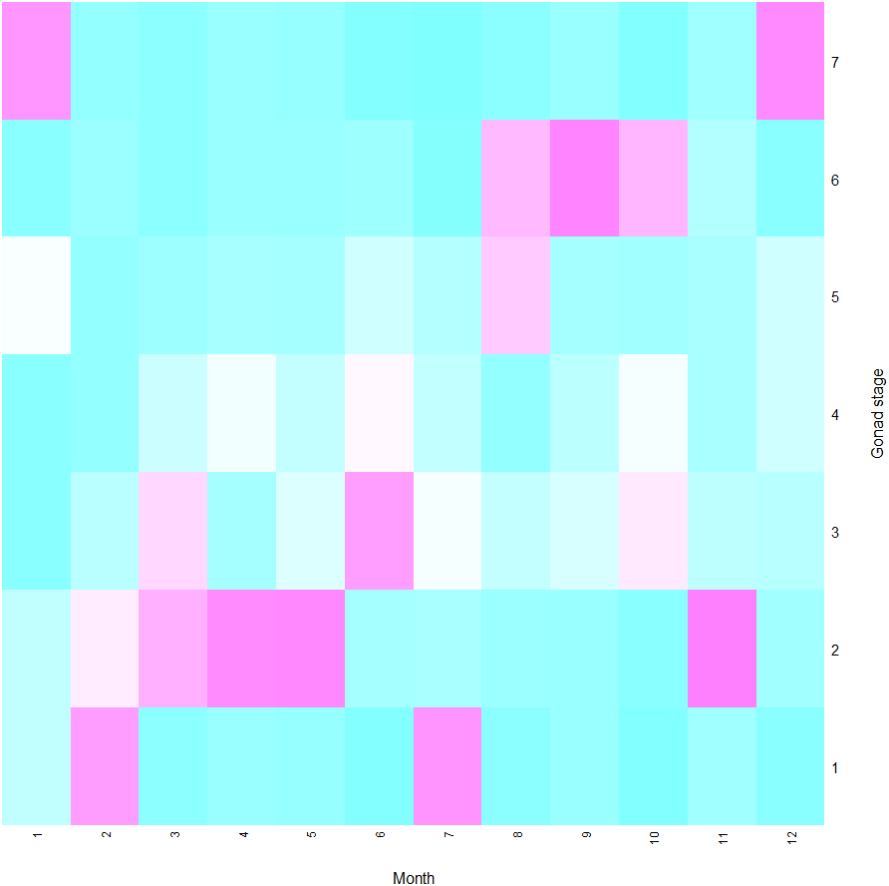
Heatmap of gonad stages of female *Procambarus clarkii* in different months. As shown in color key, white color represents absent and purple represents the highest occurrences. Samples were eight for January, 48 for February, 156 for March, 94 for April, 118 for May, 127 for June, 147 for July, 106 for August, 81 for September, 78 for October, 30 for November, and 19 for December, respectively.

The proportions of different ovarian stages across the year are shown in Fig. 4. In February, ovaries with stage I were present at maximum abundance, and ovaries with stage II increased until May. In June, the percentage of ovaries with stage III was the highest. In July, although ovaries with stage IV increased, the proportion showed only a slight increase due to more juveniles with stage I occurrence. The proportion of ovaries with stage V peaked in August. Most female crayfish ovaries developed to stage VI from August to October, with a peak in September. In November, the proportion decreased dramatically and ovaries with stage II dominated. From December to January, most ovaries developed to stage VII after spawning.

**Figure 5:**
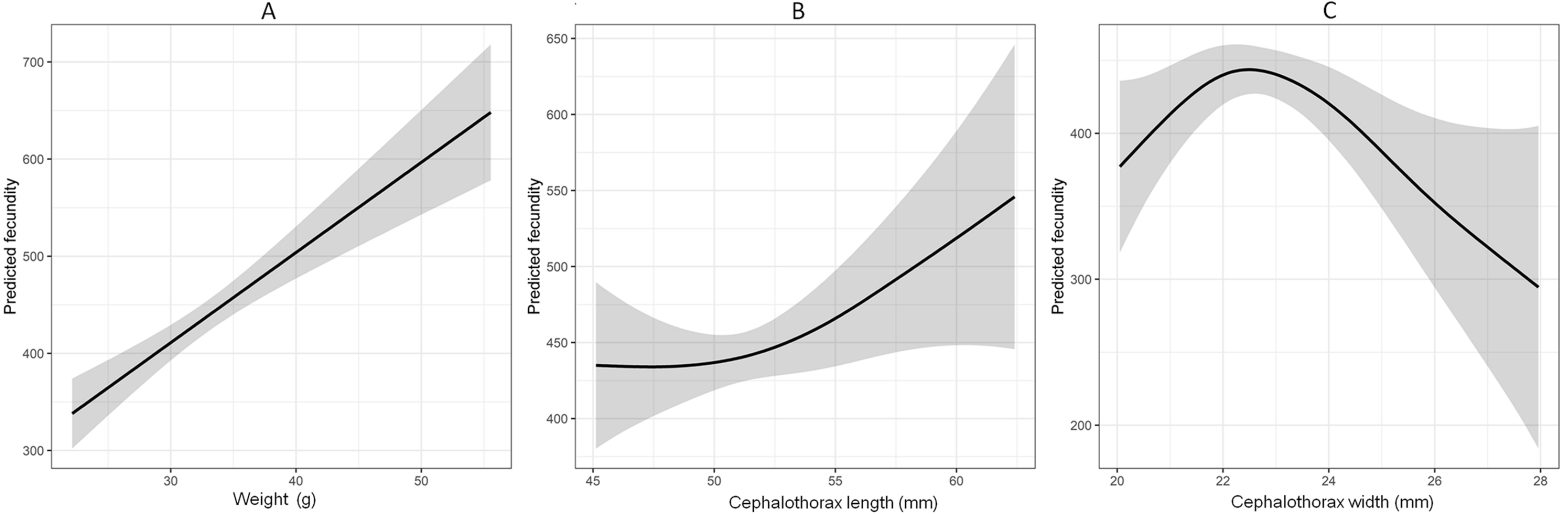
Generalized additive model (GAM) explaining the relationship between weight, cephalothorax length, cephalothorax width and fecundity of *Procambarus clarkii*. Solid lines represent the estimated smooth function and the grey areas represent the 95% confidence interval.

Fecundity was only assessed from September to December. The relationships of weight, CTL, cephalothorax width and fecundity explained by the GAM model are shown in Fig. 5. The model explained the 76.6% of total deviance, with a high value of *R*^2^ = 0.745. The relationship between weight and fecundity was approximately linear, indicating the fecundity increased with increasing weight (*F* = 36.72, *P* < 0.001). Although increased with cephalothorax width at the beginning, the fecundity was at the onset of decrease after 23 mm of the cephalothorax width (*F* = 3.80, *P* = 0.006). The fitted curve for CTL was slightly concave based on the interpretation of the GAM plots, however, there was no evidence of interactions observed (*F* = 1.97, *P* = 0.16). The average number of eggs berried per female crayfish was 429 ± 9, with the minimum and maximum value of 290 and 610, respectively.

### Population dynamics parameters estimates

**Figure 6:**
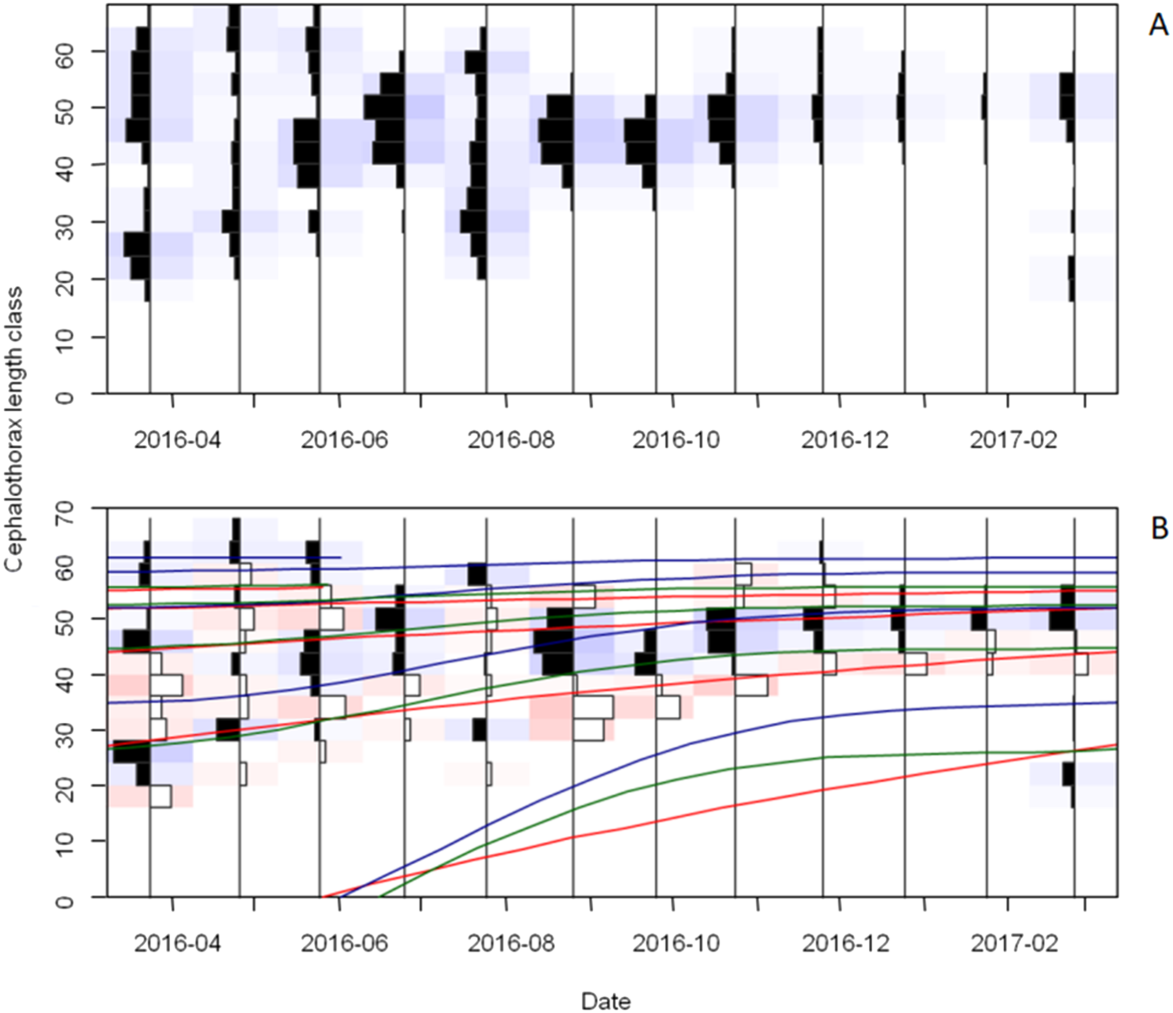
Cephalothorax length (CTL) frequency distribution and growth curves of female *Procambarus clarkii* during sampling time. CTL frequency data visualized in terms of catches (A) and restructured data (B) with a moving average setting of MA = 5. Graphical fit of estimated and true growth curves plotted through the CTL frequency data. The growth curves with the true values are displayed in red, while the blue and green curves represent the curves of ELEFAN_SA (Electronical length frequency analysis-simulated annealing, estimate growth paramaters with simulated annealing) and ELEFAN_GA (estimate growth paramaters with genetic algorithm), respectively. Positive (black) and negative (white) scored bins are indicated by the histogram direction.

**Figure 7:**
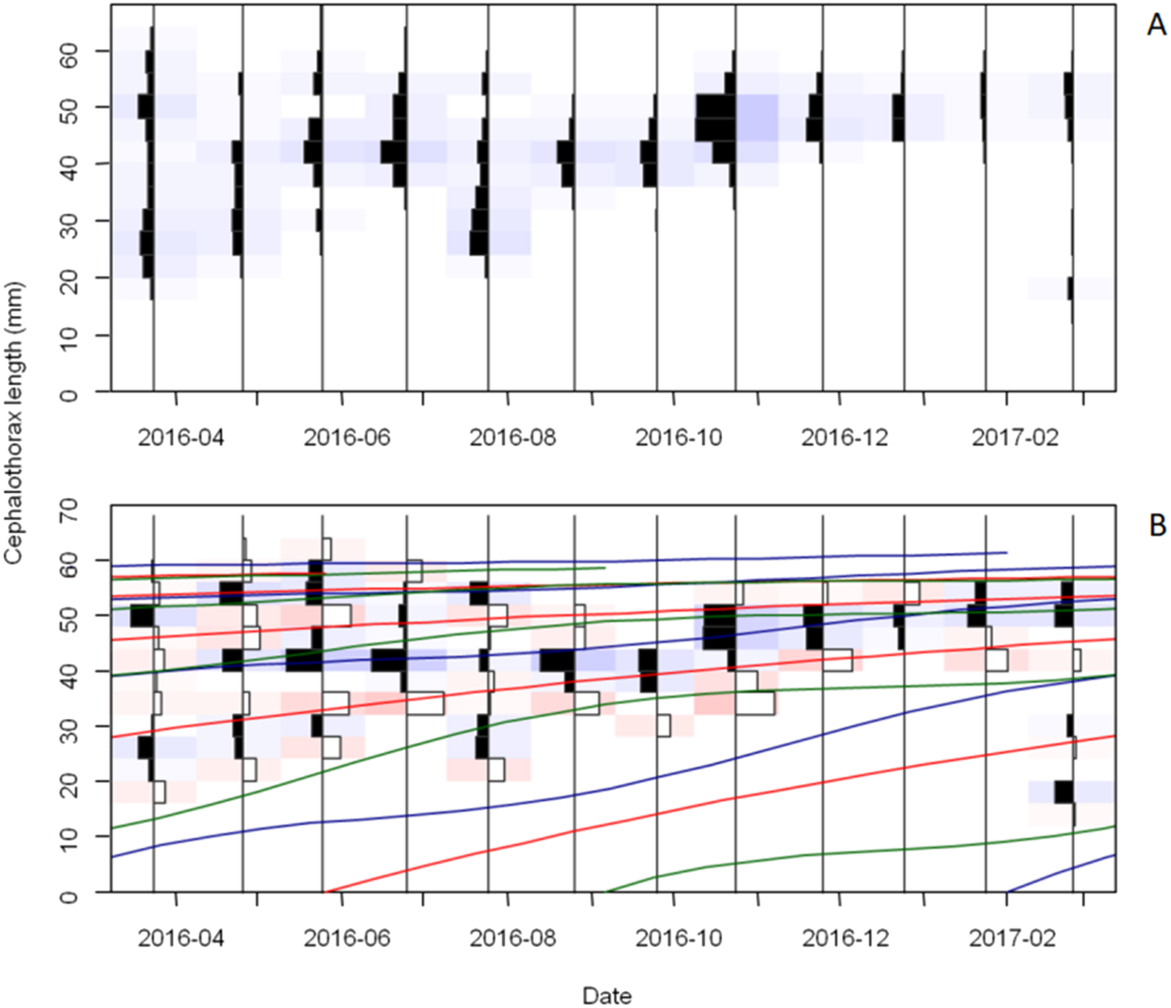
Cephalothorax length (CTL) frequency distribution and growth curves of male *Procambarus clarkii* during sampling time. CTL frequency data visualized in terms of catches (A) and restructured data (B) with a moving average setting of MA = 5. The graphical fit of estimated and true growth curves plotted through the CTL frequency data. The growth curves with the true values are displayed in red, while the blue and green curves represent the curves of ELEFAN_SA (Electronical length frequency analysis-simulated annealing, estimate growth parameters with simulated annealing) and ELEFAN_GA (estimate growth parameters with genetic algorithm), respectively. Positive (black) and negative (white) scored bins are indicated by the histogram direction.

The frequency distributions of monthly CTL (distinguished by sexes) and the growth curves, fitted by ELEFAN using “TropFishR” package, are presented in Figs. 6A, 6B and 7A, 7B. The CTL data of collected crayfish was classified into 17 size classes of 4 mm interval size classes. From the analysis of the CTL frequency distributions, five growth cohorts were observed for both females and males, each cohort corresponding to 1 size class.

For females, the growth curves highlighted five cohorts (Fig. 6B). In the first cohort, offspring released from May 2016 had about 25 mm CTL in February 2017. For the second and third cohorts, individuals had about 28 mm and 43 mm CTL in March 2016 and reached the CTL of about 42 mm and 49 mm in February 2017. For the fourth and fifth cohorts, crayfish did not show obvious growth during the whole sampling period.

For males, there were also five growing cohorts and showed similar growth patterns with females (Fig. 7B). In the first cohort, offspring from May 2016 had about 28 mm CTL in February 2017. For the second and third cohort, individuals had about 29 mm and 45 mm CTL in March 2016 and reached the CTL of about 42 mm and 52 mm in February 2017. Crayfish composed of the fourth and fifth cohorts also did not show obvious growth.

The estimated population dynamics parameters (*L*_*inf*_, *K*, *t*_0_, and *t*_*max*_, *Z*, *M*, *F*, and *E*) for both females and males during the sampling period are shown in Table 1. Although females had higher values of *K* and *M*, results of pairwise Wilcox test showed no significant differences in those parameters between sexes (*P* = 0.19).

**Table 1:** Von Bertalanffys parameters of the studied *Procambarus clarkii* population and others from Europe reported in previous studies.

Sex	*K* (year^−1^)	*L*_*inf*_ (mm)	*t*_0_ (year)	*t*_*max*_ (year)	Ø′ (year^−1^)	*Z* (year^−1^)	*M* (year^−1^)	*F* (year^−1^)	*E*	References
Male	0.81	60.93	−0.29	3.41	8.01	2.32	0.93	1.39	0.60	Present study
Female	0.86	58.12	−0.27	3.22	7.97	1.93	1.02	0.91	0.47	
Male	0.340	68.25	−0.110	8.71	3.19	3.43	1.14	2.29	0.67	Maccarrone et al. (2016)
Female	0.350	67.20	−0.260	8.31	3.19	3.83	1.16	2.67	0.70	
Male	0.59	69.35	−0.09	5.08		5.50	2.83	2.67	0.49	Dörr & Scalici (2013)
Female	0.58	73.71	−0.14	5.17		5.10	2.77	2.33	0.46	
Male	0.49	74.60	−0.022	6.1	3.44	2.26	2.26	0.00	0.00	Chucholl (2011)
Female	0.45	79.80	−0.027	6.6	3.46	2.79	2.55	0.24	0.09	
Male	0.33	68.3	−0.37	8.73		2.88	1.63	1.25	0.43	Scalici et al. (2010)
Female	0.32	74.6	−0.43	8.95		3.11	1.77	1.34	0.43	
Male	0.69	62.71	−0.1	4.25	3.43	2.99				Scalici & Gherardi (2007)
Female	0.68	65.52	−0.1	4.31	3.47	4.71				

**Notes.**

*L*_*inf*_ asymptotic cephalothorax length (CTL) *K* growth coefficient *t*_0_ initial condition parameter (when crayfish have CTL = 0, although biologically meaningless, it represents an important component of curve) *t*_*max*_ expected longevity Ø′ growth parameter index *Z* total mortality rate *M* natural mortality rate *F* fishing mortality rate *E* exploitation rate

## Discussion

The present study was based on a large sample size (2051 crayfish), aiming to improve the knowledge of reproduction and population dynamics of commercial *P. clarkii* population.

### Sex ratio

The overall sex ratio was near 1:1, but it varied throughout sampling months. In August and September, females were abundant and the situation was reversed from October to December, as reported by previous studies (Dörr et al., 2006; Mueller, 2007; Alcorlo, Geiger & Otero, 2008; Peruzza et al., 2015). This discrepancy in the sex ratio observed in our study was probably due to the reproductive activities of females, which tended to stay in burrows for parental care to their offspring and could be less easily trapped (Gherardi & Barbaresi, 2000; Thiel, 2000; Dörr et al., 2006; Donato et al., 2018). In addition, we also recorded an interesting phenomenon regarding the increase in proportion of males being observed during the reproductive period (September to December), likely due to the search for a mate (Peruzza et al., 2015).

In order to maximize short-term catch rates and profitability, farmers intentionally target particular sizes or sex of crayfish during catching periods (Zhou et al., 2010). For example, due to the low catch rates and reproductive activities of females, more male crayfish are selectively harvested during the reproductive period. This males-directed selectivity may impose adverse effects on reproductive output since it causes difficulties in females finding mates. Similar cases were also found in crabs (Gray & Powell, 1966; Smith & Jamieson, 1991). Thus, in fishery management, the possible side effects of sex selection on reproductive success of the population should be considered (Zhou et al., 2010).

### Reproductive pattern analysis

In the present study, spawning activities of female *P. clarkii* mostly took place from September to November. However, several ovigerous females were also caught from March to May, which suggested the possibility of two recruitment phases yearly (March to May and October to November). This was also confirmed by the characteristics of the samples collected in spring, where the release of larvae frequently took place. We inferred that those females had most likely laid eggs at the end of the previous autumn. This was because eggs development was strongly linked to water temperature and previous studies showed that it would take up to 130 days until eggs hatching at the temperature below 10 °C (Suko, 1954; Suko, 1956). During our study, the mean temperature was 13.58 °C and 10.03 °C for November and December, which suggested that eggs in late autumn were probably prevented from hatching by low water temperature. Those eggs, having survived the harsh winter conditions, would be more likely to hatch in the next spring when the environment is favorable. Accordingly, we found crayfish larvae in spring. Thus, delaying hatching could be an adaptive strategy of *P. clarkii* for unfavorable environmental conditions such as low water temperature in winter (Lass et al., 2005).

In this study GSI increased rapidly from July reaching its peak in September and then diminished from October, and was near to the lowest value in November. This indicated that crayfish spawning initiated in September and was achieved in November. Although February encountered a slight increase in GSI values, we speculated that it might be attributed to the small sampling sizes since most crayfish slowed down their activities and were hard to catch due to low water temperature (Rodríguez, Bécares & Fernández-Aláez, 2003).

In some places, different recruitment events were found per year. For instance, there were two-yearly distinct recruitments in Italy (Scalici & Gherardi, 2007; Maccarrone et al., 2016), southern Portugal (Adao & Marques, 1993), Spain (Cano & Ocete, 1997; Alcorlo, Geiger & Otero, 2008), America (Sommer, 1984) and Japan (Suko, 1958), while one main recruitment occurred in central Portugal (Anastácio & Marques, 1995) and Germany (Chucholl, 2011). The differences in plastic recruitment patterns were difficult to explain, because gonad development and eggs incubation depended on different environmental features, such as water temperature, habitat uses, and food resources (Sastry, 1983; Harlioğlu & Farhadi, 2017). In our study, the single spawning peak with two recruitment patterns is most likely driven by the low water temperature, but further studies are still needed to test it.

Generally, the fecundity of crustaceans is correlated with females’ body sizes or weight, and it shows variability in different populations (Harlioğlu et al., 2004; Nakata & Goshima, 2004). Our study accords with those findings. The strong linear relationship between weight and fecundity indicated that heavier females tended to produce more eggs. Similar results have been reported for other crustaceans such as *Cherax quadricarinatus* (Öndes, Kaiser & Murray, 2017) and *Oziothelphusa senex senex* (Swetha, Girish & Reddy, 2015). Moreover, it was noteworthy that fecundity started to decrease when cephalothorax width was over 23 mm. Actually, this result was in contrast from what were reported in several previous studies, which showed fecundity always increased with the increasing cephalothorax width (Lizarraga-Cubedo et al., 2003; Hamasaki, Fukunaga & Kitada, 2006; González-Pisani & Greco, 2014). We inferred that the declined fecundity was mostly due to the onset of senescence of larger females, and thus resulting in lower relative reproductive output (Sudha & Anilkumar, 1996). Furthermore, the average fecundity of *P. clarkii* in the current study was much higher than those in Germany (Chucholl, 2011) and its native range (Penn, 1943), but similar to that in Kenya (Oluoch, 1990). Such differences in fecundity could be explained by the different female sizes or the temporal variations in food availability for different populations (Beyers & Goosen, 1987). In our study, favorable environment such as abundant food resources (e.g., artificial diet) could result in large young females and thus higher reproductive output (Alcorlo, Geiger & Otero, 2008).

### Population dynamics parameters estimates

Length-frequency analysis showed the structure of commercial *P. clarkii* population constituted of five cohorts for both females and males. The second and third cohorts were constituted of abundant younger crayfish, which were fast-growing individuals. Actually, cohorts of *P. clarkii* varied considerably in numbers across populations. For example, there were five cohorts in Portugal (Anastácio et al., 2009), six in Italy (Dörr & Scalici, 2013), seven in China (Huang et al., 2012), and eight and nine for males and females in Germany (Chucholl, 2011). It was easy to observe differences in CTL sizes of *P. clarkii* among those studies. We inferred that the differences were mainly attributed to trapping activities. In our study, only crayfish with a CTL higher than 15.20 mm were captured, which could be caused by the selectivity of sampling traps used in studies. Therefore, it was possible that the CTL frequency analysis only partially described the real population structure.

Comparing with previous studies on Von Bertalanffy’s growth parameters of *P. clarkii* showed that the *L*_*inf*_ in our study was smaller than others (Table 1, Scalici et al., 2010; Chucholl, 2011; Dörr & Scalici, 2013; Maccarrone et al., 2016). We speculated that it could be related to density-dependent growth. Generally, higher density leads to a decline in resources availability, which consequently could result in a decrease in *L*_*inf*_ (Svedäng & Hornborg, 2014). The *K* and *Ø*′ obtained for females and males in our study were higher, which suggested that the *P. clarkii* in our studied area maintained a relatively high growth rate. The variability in growth rates of *P. clarkii* may relate to several ecological factors, especially temperature and nutrition (Dörr & Scalici, 2013). The optimal temperature for *P. clarkii* growth is approximately 23 °C and low temperatures at higher latitudes in the previous studies probably lead to slow growth rates (Espina & Herrera, 1993). Crayfish growth is highly correlated with nutrition, and the high food availability and nutrition-sufficiency of artificial diet in our study could guarantee *P. clarkii* better growing conditions than the wild ones.

Our findings showed that fishing mortality rate *F* of male *P. clarkii* was higher than females, which indicated that males were under high fishing pressure (0.91 and 1.39 year^−1^ for females and males, accounting for 47% and 60% of *Z*, respectively). We inferred that this was related to the males-directed fishing selection during the reproductive period. In our study, high proportions of males were captured during reproduction. This fishing selection generally causes damage and stress to males, which has negative effects on their growth and survival (Chopin & Arimoto, 1995). Even though some crayfish escape from fishing, they may be injured and die later due to physical damage, which might account for the elevated mortality in our study. *M* is related to many factors except fishing, such as predation and starvation. For the aquaculture practice, sufficient nutrition supplies and farmers’ efforts to eradication other unwanted fish or crayfish guarantee crayfish under very low starvation and predation pressure in our study, which could explain why *M* is lower than that of wild populations (Table 1). Generally, *M* has been widely used as the upper limit of *F* for sustainable fishing, which suggests that *E* should be less than 0.5 to prevent populations from overfishing (Gulland, 1971; Gulland, 1983; MacCall, 2009; Froese et al., 2016). The estimated *E* of 0.60 for males in our study was higher, indicating that the male *P. clarkii* was overexploited and under high fishing pressure. In such a situation, the fishing activities should be well monitored to protect the commercial *P. clarkii* population from further depletion.

### Implications for aquaculture management

In recent years, *P. clarkii* has become one of the most important freshwater products in China, and the market demands greatly exceed aquaculture supplies. As the males *P. clarkii* have been overexploited, efforts to improve productivity and sustainability of the crayfish population are crucial for the aquaculture management. Therefore, balanced exploitation should be encouraged to alleviate fishing mortality arising from unsustainably fishing activities and increase the overall sustainability of *P. clarkii* populations (Svedäng & Hornborg, 2014). Based on the results of our study, we suggest reducing fishing pressure for commercial *P. clarkii* population through the following two parts.

First, we suggest reducing the fishing intensity on immature crayfish before they reach maximum sizes. In our study, the second and third growth cohorts were made of abundant fast-growing individuals while crayfish of the fourth and fifth growth cohorts showed extremely slow growth. For sustainable exploitation scenario, reducing fishing on younger crayfish and selectively catching old crayfish with slow growth or small sizes will help to promote large-sized individuals and render crayfish culture more profitable. These old crayfish can be distinguished by CTL sizes (more than 50 mm, Figs. 6B and 7B) and maturity (Penn, 1943; Cang, Miltner & Avault, 1982; Jarboe & Romaire, 1995; Taketomi, Murata & Miyawaki, 1990; Taketomi, Nishikawa & Koga, 1996). This would also offer more access to environmental resources (e.g., food availability) for juveniles and then may increase growth rates.

We also suggest reducing the fishing intensity and avoiding sex selection during the reproductive phase of *P. clarkii*. Trapping is a widespread method for management and is considered to be highly efficient especially for higher crayfish sizes. However, this high efficiency is achieved only when the trapping activity is conducted for a proper period of time. The high fishing pressure during the reproductive season could have negative effects on reproductive potentials, and then influence long-term stock productivity (Van Overzee & Rijnsdorp, 2015). Furthermore, fishing may also cause the death of offspring. Thus, restricting fishing pressure on spawning crayfish would be an effective measure to enhance reproductive output and promote population productivity. In our study, more male crayfish were harvested during the reproductive period, which may cause difficulties in females finding mates, and thus affect reproductive success. Based on our findings that the spawning activities and the main recruitment occurred from October to November, we suggest reducing fishing pressure and avoid male selection during this period.

## Conclusions

This study was conducted to determine the reproductive pattern and population dynamics of commercial *P. clarkii* population. The spawning activities of female *P. clarkii* took place from September to November. There were two recruitments yearly, with a major one from October to November and the minor one from March to May. There were five growth cohorts for females and males, with higher growth rates than previous studies. Males *P. clarkii* were overexploited and under high fishing pressure, as evidenced by a high exploitation rate of 0.60 for males. Our findings thus suggest reducing fishing intensity on immature crayfish and avoid male selection during the reproductive phase to improve aquaculture sustainability. With this study, we hope to encourage further works on commercial crayfish stock assessment and management to promote population productivity and sustainable fisheries.

##  Supplemental Information

10.7717/peerj.6214/supp-1 Figure S1 Micrograph of seven ovarian development stages of *Procambarus clarkii* Figure (A–G) show the histological changes for ovarian stage I VII.

10.7717/peerj.6214/supp-2 Table S1 Raw data for the study

10.7717/peerj.6214/supp-3 Supplemental Information 1 Data code for software R

## Additional Information and Declarations

### Data Availability

The following information was supplied regarding data availability: The raw data and code are provided in the Supplemental Files 2 and  3.
