# Supplementary figures and images for: Reproductive pattern and population dynamics of commercial red swamp crayfish (Procambarus clarkii) from China: implications for sustainable aquaculture management
Source: PeerJ. 2019 Jan 23;7:e6214. doi: 10.7717/peerj.6214 (PMC6347965; doi:10.7717/peerj.6214)

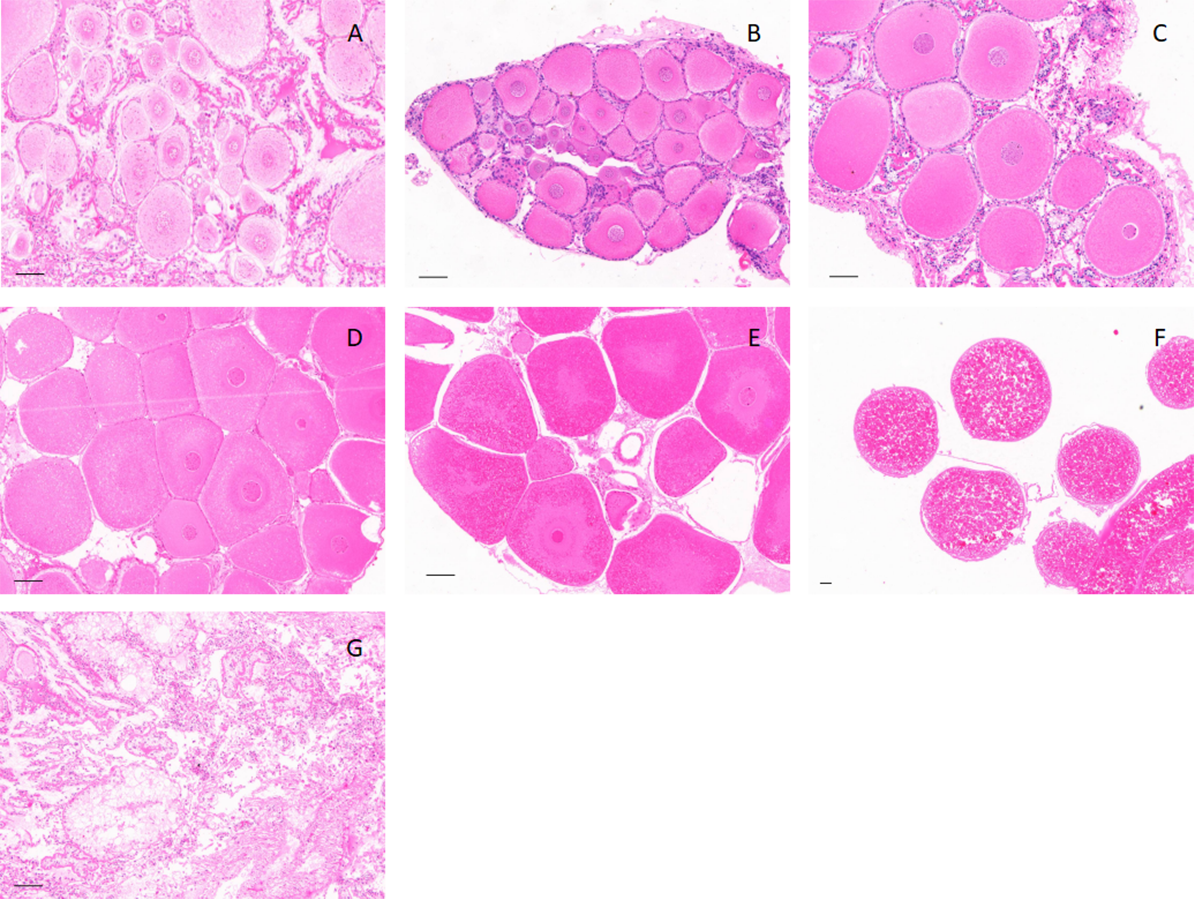

Supplement: Figure S1 — Figure (A–G) show the histological changes for ovarian stage I VII. [file peerj-07-6214-s001.png]
